# Exploring the Role of AI in Enhancing Nuclear Medicine Report Impressions Generated by Trainees and ChatGPT-4o: Comparative Evaluation Study

**DOI:** 10.2196/94833

**Published:** 2026-07-08

**Authors:** Alireza Khatami, Farzad Abbaspour-Raddakheli, Christiane Wiefels, Eugene Leung

**Affiliations:** 1 Division of Nuclear Medicine and Molecular Imaging, Department of Medicine, The Ottawa Hospital, University of Ottawa, 501 Smyth Road, Ottawa, ON, K1H 8L6, Canada, 1 613-798-5555 ext 71278, 1 613-737-8752; 2 Department of Medical Imaging, Division of Nuclear Medicine, McGill University Health Center, McGill University, Montreal, QC, Canada

**Keywords:** artificial intelligence, nuclear medicine, ChatGPT, large language models, report

## Abstract

**Background:**

Accurate reporting in nuclear medicine is essential for clinical decision-making. Trainees often generate preliminary reports with variable quality, and artificial intelligence (AI) tools such as ChatGPT-4o may enhance report clarity and accuracy, particularly in the impression section of the report.

**Objective:**

This study aimed to evaluate and compare the quality of positron emission tomography / computed tomography report impression sections generated by trainees and by the AI chatbot ChatGPT-4o (OpenAI), focusing on correctness, clarity, completeness, organization, use of diagnostic certainty terminology, and physician satisfaction.

**Methods:**

The impression sections of 200 positron emission tomography/ computed tomography reports generated by trainees and AI (100 reports each) were compared. The AI generated the impressions based on the stem, clinical history, and technical sections of the trainee-generated reports. Reports were blindly rated by 3 nuclear medicine physicians. Survey questions, including Likert-scale questions, assessed correctness, use of certainty terms, clarity, completeness, organization, and satisfaction. Statistical analyses included Fisher exact test, 2-tailed *t* tests, chi-square tests, ANOVA, and effect sizes (Cohen *d*). Thematic analysis was performed on free-text comments.

**Results:**

AI-generated impressions were rated as correct in 92% of reports (mean 0.92, SD 0.27) and trainee-generated impressions rated as correct in 91% of reports (mean 0.91, SD 0.29; *P*=.80, negligible effect size). AI-generated impressions included certainty terms more frequently than trainee-generated impressions (94% vs 81%, respectively; *P*=.005; *χ*²_2_=6.58, small effect size). AI also used higher-level certainty terms more frequently than trainees (mean 4.23, SD 1.25 vs mean 3.69, SD 1.93 on a 5-point scale; *P*=.02). Clarity was high in both groups (AI: mean 4.49, SD 0.70, and 89.8% of reports rated as clear vs trainees: mean 4.35, SD 0.85, and 87.0% of reports rated as clear; *P*=.20). Completeness was significantly higher for AI-generated impressions than trainee-generated impressions (mean 4.52, SD 0.76, and 90.4% complete vs mean 4.09, SD 1.01, and 81.8% complete, respectively; *P*<.001, small-to-medium effect size). Organization was similar between the groups (mean 4.22, SD 0.91 vs mean 4.39, SD 0.74; *P*=.15). Satisfaction ratings were also compared, with AI-generated impressions achieving a mean score of 4.31 (SD 0.77) and 86.2% satisfaction, compared with a mean score of 4.14 (SD 0.91) and 82.8% satisfaction for trainee-generated impressions (*P*=.16). Thematic analysis showed that trainee-generated impressions were more frequently criticized for accuracy (74% vs 19%; *P*<.001) and actionability (20% vs 4.8%; *P*=.02), whereas AI-generated impressions were more frequently criticized for excessive length (33% vs 0%; *P*<.001).

**Conclusions:**

These findings suggest that the AI chatbot ChatGPT-4o can serve as a valuable adjunct in nuclear medicine reporting, particularly at the resident level, by improving decisiveness and completeness while complementing trainee education and clinical oversight.

## Introduction

Nuclear medicine reports play a pivotal role in guiding oncologic management. Errors or variability in impressions can lead to mismanagement, unnecessary investigations, or delayed treatment. Previous studies estimate radiologic error rates to be around 30%, with real-time clinical error rates closer to 3% to 5% [1]. In nuclear medicine, accurate interpretation and communication are particularly crucial due to their role in staging, therapy assessment, and guiding biopsies. Artificial intelligence (AI), such as large language models (LLMs) including ChatGPT, has demonstrated the ability to generate structured and coherent text. Its potential application in medical reporting offers opportunities for reducing variability, enhancing completeness, and providing educational support for trainees. However, concerns remain regarding accuracy, generic phrasing, and contextual nuance [2-4].

Accurate nuclear medicine reporting is crucial for clinical decision-making, though variability in impressions, especially common in trainee-generated reports, can affect report quality. Recent advances in LLMs, like ChatGPT-4o and later versions, have shown promise in enhancing the clarity, completeness, and standardization of these reports. Multimodal LLMs trained on image-text pairs help generate clinically coherent and structured medical narratives, reducing variability and improving diagnostic confidence [5]. It has been shown that LLMs can be very useful in medical imaging. As these models become more advanced, they will be able to understand both images and text together, helping doctors get clearer and more accurate information [6]. These models facilitate automatic extraction and analysis of complex clinical data, which can help radiologists in creating clearer, more consistent reports [7]. A recent study evaluating ChatGPT-4o and comparing it with humans in diagnostic accuracy demonstrated that AI language models can support clinical reasoning and reporting quality while simultaneously supporting medical education. However, challenges in integration continue to limit their clinical implementation [8]. These applications remain subject to important clinical and ethical considerations, including limitations in accuracy, contextual interpretation, and the need for physician oversight.

This study compares AI-generated impressions with those generated by nuclear medicine trainees. The impressions were scored by 3 experts, who were blinded to the source of the impressions. The quantitative and qualitative analyses were conducted to determine whether AI can complement or enhance the reporting process in nuclear medicine.

## Methods

### Ethical Considerations

This study was approved by the Ottawa Health Science Network Research Ethics Board (OHSN-REB Protocol number 20240543-01H).

### Study Design and Setting

We performed a comparative observational prospective study at a single tertiary-care nuclear medicine division, evaluating impressions of positron emission tomography (PET)/ computed tomography (CT) reports generated between September 30, 2024 and December 29, 2024. The study compared trainee-generated impressions with impressions generated by an LLM (ChatGPT-4o) from identical trainee-generated report stems before attending physician review and final report sign-off.

### Population and Data Source

We gathered 200 PET/CT reports and deidentified them. Each report consisted of a clinical history, technical aspects, key findings (stem), and a final impression. The trainees had access to clinical notes, pathology reports, and prior imaging findings when drafting their reports and impressions during routine reporting, prior to review by attendings. The first 3 sections of the 200 PET/CT reports were completed by trainees. Half of the cases had the impressions generated by the trainees, whereas the remaining half (100 cases) had ChatGPT-4o–generated impressions.

The AI-generated report was produced using a standardized prompt provided to ChatGPT-4o. A project was defined within the application, and relevant templates were uploaded along with a structured prompt. The prompt specified how the impression should be organized based on the findings described in the stem, reason for referral, and any additional information available at the time of reporting. All scans were selected consecutively during routine reporting. The selection included a random mixture of PET/CT to reflect a representative sample of clinical practice, including F18-FDG (fluorine-18 fluorodeoxyglucose), Ga68-DOTATATE, and Ga68-PSMA (gallium-68 prostate-specific membrane antigen) PET/CT images. All identifiers were removed, and alphanumeric codes were assigned and aggregated into a Microsoft Excel file formatted to collect impression quality scores by the reviewers.

### AI Impression Generation

To limit output variability and ensure that the generated content was clinically relevant and appropriate, we developed a project template for ChatGPT-4o. The template instructed the model to create an organized and structured impression by (1) synthesizing the history, requisition, and PET/CT findings into a concise but complete impression (≤3 sections); (2) using a certainty lexicon mapped to probability categories (very low <10%, low ~25%, intermediate ~50%, high ~75%, and highest >90%); (3) including a diagnosis or differential; (4) suggesting appropriate next steps (eg, additional imaging, histopathology correlation, clinical consult, or interval follow-up); and (5) providing staging or response assessment where applicable (eg, complete response, partial response, Deauville score for lymphoma, TNM, molecular imaging TNM, or Krenning score where relevant). We performed a pilot study with a limited number of random cases prompted on lymphoma, lung cancer, and Ga68-DOTATATE and refined the instructions to ChatGPT-4o on how to create better, more comprehensive, yet short impressions before unifying all prompts and instructions into a single project (“Impression for PET-CT Reports”). The final prompt and certainty lexicon were held constant across cases. These categories were selected to reflect commonly used probability-based terminology in radiology and nuclear medicine reporting. The questionnaire and a list of certainty words were provided to raters (Multimedia Appendix 1).

### Rater Panel and Blinding

Three board-certified nuclear medicine physicians (coded as EL, CW, and FA-R) independently rated each impression. Raters were blinded to the report origin (AI vs trainee) and to the trainee subgroup (residents vs fellows). Two raters reviewed 65 impressions, and 1 rater reviewed 70 impressions, with sufficient overlap to conduct interrater agreement analysis. Raters’ evaluations were solely based on the information in the first 3 sections of the report, and no imaging or clinical data were reviewed.

### Questionnaire and Outcome Measures

Each rater used a predefined questionnaire encoded in an Excel file to rate the impression regarding the following aspects: (1) correctness or relevance of the impression (binary scale scored −1, 0, 1; reported as proportion correct); (2) presence of at least one certainty word (yes or no); (3) certainty category (0‐5 scale: 0=none, 1=very low to 5=highest); (4) Likert-scale (1-5) rating questions regarding clarity, completeness, organization, and overall satisfaction; (5) presence of an action plan and type (further imaging, histopathology, close follow-up, and clinical consult; multiple responses permitted); and (6) free-text comments for thematic analysis. The questionnaire was internally reviewed and pilot-tested using a limited set of PET/CT cases before formal evaluation. Correctness was defined as the factual accuracy of the impression relative to the findings section of the same report, ensuring that the interpretation was consistent with the imaging descriptions. Relevance was defined as the clinical pertinence and prioritization of information within the impression, reflecting whether it addressed key diagnostic questions and actionable conclusions. Both attributes were assessed by expert reviewers using a structured 5-point Likert questionnaire based solely on the written report content. Similarly, clarity was defined as the degree to which the impression communicated its diagnostic message in a precise, unambiguous, and easily understandable manner. Reviewers assessed clarity using a 5-point Likert scale (1=very unclear to 5=very clear), considering sentence structure, readability, terminology, and logical flow of the impression text. Raters also evaluated the action plan based on information provided in the stem of the report and the referral note. If the action plan provided was inappropriate, the rater marked it appropriately and was able to comment on it in the open-ended comment section. Completeness was defined as the degree to which the impression summarized all major findings and directly addressed the primary clinical question. Reviewers rated completeness subjectively on a 5-point Likert scale, considering whether any omission could alter clinical interpretation or management. Examples of incomplete impressions included failure to mention key lesions or to provide a definitive diagnostic statement when sufficient information was available. Finally, the rater identifier was also recorded for interrater analysis.

### Statistical Analysis

Interrater reliability was assessed using Fleiss *κ* on a common sample, with *κ*=0.61 to 0.80 interpreted as substantial agreement. Continuous outcomes (Likert means) were compared using independent-samples 2-tailed *t* tests (AI vs trainees), within subgroup comparisons (AI vs residents vs fellows), and using *t* tests and/or one-way ANOVA as appropriate with related *P* values. Categorical outcomes were compared with chi-square tests and reported with *P* values and Cramér V where applicable. Effect sizes for continuous outcomes were expressed as Cohen *d* (0.2 for small, 0.5 for medium, and 0.8 for large). Statistical significance was set at α=.05 (2-sided). All descriptive and comparative statistical analyses (means, SDs, *t* tests, and chi-square tests) were computed in Microsoft Excel. Advanced analyses, including one-way ANOVA with Tukey Honestly Significant Difference post hoc testing, were performed in GraphPad Prism (version X). Cohen *d* effect sizes were calculated manually from group means and pooled SDs using standard formulae.

## Results

### Demographics and Interrater Reliability

We analyzed 200 impressions, comprising 100 (50%) AI-generated and 100 (50%) trainee-generated impressions. Of the 100 scans, 90 (90%) were F18-FDG, 7 (7%) were Ga68-DOTATATE, and 3 (3%) were F18-DCFPyL (prostate-specific membrane antigen) PET/CT scans. Of the 100 cases, 34 (34%) were lung cancer, 19 (19%) lymphoma, 8 (8%) breast cancer, 8 (8%) melanoma, 7 (7%) neuroendocrine tumor, 7 (7%) colon cancer, 5 (5%) esophageal cancer, 3 (3%) prostate cancer, 3 (3%) multiple myeloma, 3 (3%) nasopharyngeal cancer, 2 (2%) pancreatic cancer, and 1 (1%) sarcoma case. Among the trainee-generated reports, 26 (26%) were authored by 2 Canadian radiology residents (referred to as “residents”), who were native English speakers and participated during their nuclear medicine rotations in postgraduate year 4 and year 5 with limited prior nuclear medicine experience. The remaining 74 (74%) reports were authored by 4 international nuclear medicine fellows (referred to as “fellows”), who were nonnative English speakers but had prior experience in PET/CT reporting during their nuclear medicine or radiology residency training, or had completed at least 1 year of nuclear medicine fellowship in their home country. Of these fellows, 2 were in year 1 and 2 were in year 2 of their current training. Rater contribution was balanced across the 200 total reviews: EL reviewed 65 (32.5%), CW reviewed 65 (32.5%), and FA-R reviewed 70 (35%). Interrater reliability was performed over 20 cases with the overlap cases used for interrater reliability assessment, including 12 of 20 (60%) cases, yielding Fleiss “*κ*” value of 0.71, which indicates substantial agreement among raters. Although the overlap sample was limited, this level of agreement suggests consistent application of the rating criteria.

### Comparative Performance

#### AI vs Trainees Analysis

**Figure 1.**
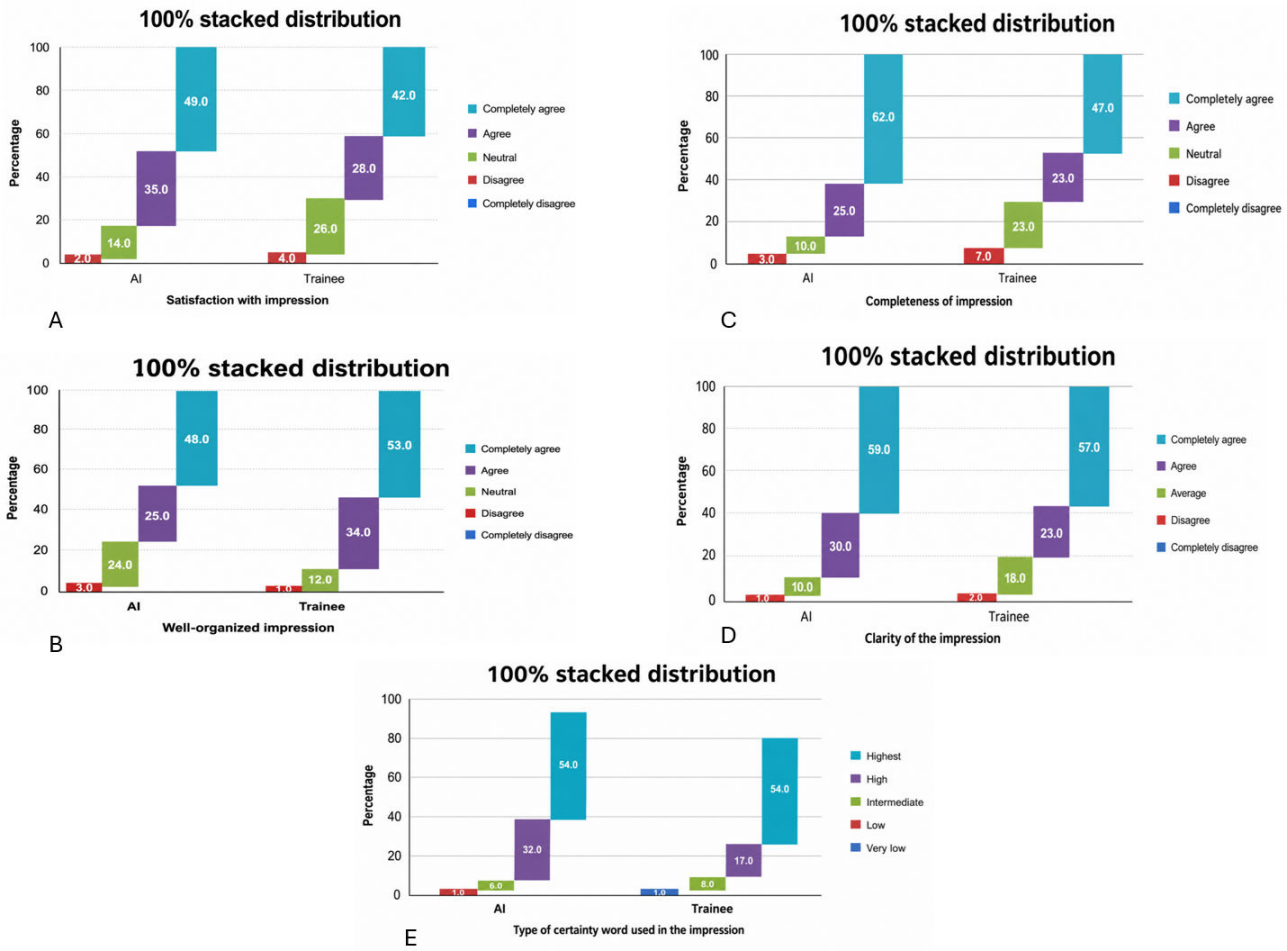
Likert-scale ratings bar chart comparing artificial intelligence (AI) vs trainee performance across domains in the impression: (A) satisfaction, (B) well-organized, (C) completeness, (D) clarity, and (E) the type of certainty word used in the impression. This includes only reports with at least one certainty term.

Primary comparative outcomes comparing AI-generated and trainee-generated impressions are detailed in Table 1, and Likert score rating distributions are summarized in Figure 1.

**Table 1.** Summary of artificial intelligence (AI)–generated impression performance metrics compared with trainee reports.

Metric	AI^a^, n=100	Trainee^a^, n=100	*t* (*df*)	*P* value	Effect size
The correctness and relevance of the information provided in the impression in relation to the findings	92% (0.27)	91% (0.29)	0.25 (198)	.80	0.036 (negligible)
At least one certainty word is used in the impression	94% (0.24)	81% (0.39)	2.82 (198)	.005	0.400 (small)
The kind of certainty word is used in the impression (0‐5 scale)	4.23 (1.25)	3.69 (1.93)	2.35 (198)	.02	0.332 (small)
The report impression was clear (0‐5 scale)	4.49 (0.70)	4.35 (0.85)	1.27 (198)	.20	0.180 (negligible)
The report impression was complete (0‐5 scale)	4.52 (0.76)	4.09 (1.01)	3.41 (198)	.001	0.483 (small)
The impression was well organized (0‐5 scale)	4.22 (0.91)	4.39 (0.74)	−1.46 (198)	.15	–0.206 (small)
Satisfaction with the impression (0‐5 scale)	4.31 (0.77)	4.14 (0.91)	1.42 (198)	.16	0.201 (small)

a Values are shown as mean (SD) for 5-point Likert metrics and as percentages (SD) for binary outcomes.

Correctness or relevance was similarly high in both groups with respective mean scores of 0.92 (SD 0.27, 92% correct) and 0.91 (SD 0.29, 91% correct; *t*_198_=0.25; *P*=.80). AI impressions more frequently contained one or more certainty terms compared to trainee impressions (94% vs 81%; *t*_198_=2.82; *P*=.005; small effect size) and used higher-level certainty term on average score (4.23, SD 1.25 vs 3.69, SD 1.93; *t*_198_=2.35; *P*=.02; small effect size). Regarding report completeness, 97% of AI-generated impressions were rated as complete compared to 92% for trainees (Figure 1A). Likewise, completeness scores were higher for AI (mean 4.52, SD 0.76 vs mean 4.09, SD 1.01; *t*_198_=3.41; *P*=.001; small effect size). Indeed, the clarity of the impression (mean 4.49, SD 0.70 vs mean 4.35, SD 0.85) and the satisfaction score (mean 4.31, SD 0.77 vs mean 4.14, SD 0.91) were slightly higher for AI but did not reach statistical significance. In contrast, impression organization scores were higher for trainees compared to AI (mean 4.39, SD 0.74 vs mean 4.22, SD 0.91) but again did not reach statistical significance.

#### Subgroup Analysis (AI vs Residents vs Fellows)

We extended the 3-group comparison among AI, residents, and fellows by analyzing continuous outcomes using one-way ANOVA with Tukey Honestly Significant Difference post hoc tests. Binary outcomes were evaluated using 3-group chi-square tests, followed by pairwise chi-square or Fisher exact tests, as appropriate.

A summary of AI-generated impression performance metrics compared with reports generated by residents and fellows is provided in Table 2.

**Table 2.** Summary of artificial intelligence (AI)–generated impression performance metrics compared with reports generated by residents and fellows.

Metric	AI^a^, n=100	Residents^a^, n=26	Fellows^a^, n=74	Overall test (*df*)	*P* value
The correctness and relevance of the information provided in the impression in relation to the findings	92 (92%)	22 (84.6%)	69 (93.2%)	*χ*^2^=1.91 (2)	.39
At least one certainty word is used in the impression	94 (94%)	20 (76.9%)	61 (82.4%)	*χ*^2^=8.26 (2)	.02
The kind of certainty word is used in the impression (0‐5 scale)	4.23 (1.25)	3.58 (2.06)	3.73 (1.89)	ANOVA, *F*=2.83 (2,197)	.06
The report impression was clear (0‐5 scale)	4.49 (0.70)	4.31 (0.88)	4.36 (0.84)	ANOVA, *F*=0.86 (2,197)	.42
The report impression was complete (0‐5 scale)	4.52 (0.76)	3.85 (1.08)	4.18 (0.97)	ANOVA, *F*=7.20 (2,197)	.001
The impression was well organized (0‐5 scale)	4.22 (0.91)	4.31 (0.84)	4.42 (0.70)	ANOVA, *F*=1.23 (2,197)	.29
Satisfaction with the impression (0‐5 scale)	4.31 (0.77)	3.85 (1.05)	4.24 (0.84)	ANOVA, *F*=3.19 (2,197)	.04

a Values are shown as mean (SD) for 5-point Likert-scale metrics and as n (%) for binary outcomes.

Group descriptive findings and their significance were as follows: certainty words were used in 94 of 100 (94%) AI-generated impressions, 20 of 26 (76.9%) resident-generated, and 61 of 74 (82.4%) fellow-generated impressions. AI performed better than trainees; differences between AI and residents (chi-square test: *χ*²_1_=6.98; *P*=.008) and between AI and fellows (chi-square test: *χ*²_1_=5.85; *P=*.02) were statistically significant. The difference between residents vs fellows (chi-square test: *χ*²_1_=0.38; *P*=.54) showed no statistical significance. There was no significant difference among the 3 groups in the level of certainty terms used. Regarding the score of impression completeness, the mean scores were 4.52 (SD 0.76; n=100) for AI, 3.85 (SD 1.08; n=26) for residents, and 4.18 (SD 0.97; n=74) for fellows. These findings likely reflect differences in prior nuclear medicine experience and PET/CT reporting exposure of fellows compared to residents. AI outperformed residents with *Δ*=0.67 (95% CI 0.21-1.14; *P*=.002) as well as fellows with *Δ*=0.34 (95% CI 0.02-0.67; *P*=.03), and both differences were statistically significant. However, the difference in performance between residents and fellows with *Δ*=0.33 (95% CI −0.15 to 0.81; *P*=.24) was not found to be statistically significant.

The mean clarity scores for the impression were 4.49 (SD 0.70) for AI, 4.31 (SD 0.88) for residents, and 4.36 (SD 0.84) for fellows. However, pairwise comparisons showed no statistically significant differences between AI and residents (*P*=.54), AI and fellows (*P*=.55), or residents and fellows (*P*=.94).

There were small differences noticed in the organization of the impression. The mean scores were 4.22 (SD 0.91) for AI, 4.31 (SD 0.84) for residents, and 4.42 (SD 0.70) for fellows. However, the differences between AI and residents (*P*=.88), AI and fellows (*P*=.26), and residents and fellows (*P*=.83) were found to be not statistically significant.

On the other hand, regarding satisfaction with impression scores, the results were as follows: for AI, the mean score was 4.31 (SD 0.77); for residents, it was 3.85 (SD 1.05); and for fellows, it was 4.24 (SD 0.84). AI outperformed residents with *Δ*=−0.46 (95% CI −0.90 to −0.03; *P*=.03); however, the comparisons between AI and fellows with *Δ*=−0.07 (95% CI −0.37 to 0.24; *P*=.86) and between residents and fellows with *Δ*=0.40 (95% CI −0.05 to 0.85; *P*=.10) were not statistically significant.

Correctness or relevance rates for impressions were 92% (92/100) for AI, 84.6% (22/26) for residents, and 93.2% (69/74) for fellows. Statistical analyses of AI vs residents (Fisher exact test, *P*=.27), AI vs fellows (chi-square, *P*=.76), and residents vs fellows (Fisher exact test, *P*=.20) showed no statistically significant differences.

The overall 3-group analyses showed that the differences in the use of certainty words in the impression (chi-square: *χ*²_2_=8.26; *P*=.02), completeness of the impression (ANOVA: *F*_2,197_=7.20; *P*=.001), and satisfaction with the impression (ANOVA: *F*_2,197_=3.19; *P*=.04) were found to be statistically significant, favoring AI performance.

However, correctness or relevance of impression (chi-square test: *χ*²_2_=1.91; *P*=.39), clarity of impression (ANOVA: *F*_2,197_=0.86; *P*=.42), organization of the impression (ANOVA: *F*_2,197_=1.23; *P*=.29), and certainty level (0‐5 scale; ANOVA: *F*_2,197_=2.83; *P*=.06) did not meet the criteria for statistical significance.

The 3-group analysis clarified differences observed in the 2-group comparison. AI-generated reports were more likely to include certainty language and achieved higher mean certainty levels. Completeness differed significantly across groups (ANOVA: *P*<.001), with Tukey tests showing AI exceeding both residents and fellows, and fellows exceeding residents numerically, likely reflecting greater prior nuclear medicine reporting experience among fellows. Satisfaction also differed (ANOVA: *P*=.04), driven primarily by higher AI ratings versus trainees. Clarity and organization did not differ significantly across the 3 groups.

An action plan was mentioned in 67 of 100 (67%) AI reports, 8 of 26 (30.7%) residents' reports, and 30 of 74 (40.5%) fellows’ reports. This difference may relate to the referral context, such as cases with already confirmed pathology or action plans provided in referral notes. However, when an action plan was expected, AI reports included one in 67 of 81 (82.7%) cases, compared with 8 of 15 (53.3%) for residents and 30 of 49 (61.2%) for fellows. A 3-group chi-square test demonstrated a significant difference (*χ*²_2_=10.18; *P*=.006), and this finding was confirmed using Fisher exact test, which is more robust for small sample sizes (*P*=.01). Pairwise Fisher exact tests showed that AI reports were more likely to include an explicit action plan compared with residents (67/81, 82.7% vs 8/15, 53.3%; *P*=.006) and fellows (67/81, 82.7% vs 30/49, 61.2%; *P*=.02). No significant difference was observed between residents and fellows (8/15, 53.3% vs 30/49, 61.2%; *P*=.72).

A pairwise comparison of performance metrics among AI-generated, resident-generated, and fellow-generated reports is provided in Table 3.

**Table 3.** Pairwise comparison of performance metrics among artificial intelligence (AI)–generated, resident-generated, and fellow-generated reports.

Metric and contrast		Statistics	*P* value	Significance
The correctness and relevance of the information provided in the impression in relation to the findings				
AI vs residents		Fisher exact test	.27	No
AI vs fellows		*χ*^2^=0.10 (1)	.76	No
Residents vs fellows		Fisher	.23	No
At least one certainty word is used in the impression				
AI vs residents		*χ*^2^=6.98 (1)	.008	Yes
AI vs fellows		*χ*^2^=5.85 (1)	.01	Yes
Residents vs fellows		*χ*^2^=0.38 (1)	.54	No
The kind of certainty word is used in the impression (0‐5)				
AI vs residents		*Δ*=−0.65 (95% CI −1.50 to 0.19)	.10	No
AI vs fellows		*Δ*=−0.50 (95% CI −1.09 to 0.09)	.11	No
Residents vs fellows		*Δ*=0.15 (95% CI −0.72 to 1.03)	.91	No
The report impression was clear (0‐5 scale)				
AI vs residents		*Δ*=−0.18 (95% CI −0.59 to 0.22)	.54	No
AI vs fellows		*Δ*=−0.13 (95% CI −0.41 to 0.16)	.55	No
Residents vs fellows		*Δ*=0.06 (95% CI −0.36 to 0.48)	.94	No
The report impression was complete (0‐5 scale)				
AI vs residents		*Δ*=0.67 (95% CI 0.21 to 1.14)	.002	Yes
AI vs fellows		*Δ*=0.34 (95% CI 0.02 to 0.67)	.03	Yes
Residents vs fellows		*Δ*=0.33 (95% CI −0.15 to 0.81)	.24	No
The impression was well organized (0‐5 scale)				
AI vs residents		*Δ*=0.09 (95% CI −0.34 to 0.52)	.88	No
AI vs fellows		*Δ*=0.20 (95% CI −0.10 to 0.50)	.26	No
Residents vs fellows		*Δ*=0.11 (95% CI −0.33 to 0.56)	.83	No
Satisfaction with the impression (0‐5 scale)				
AI vs residents		*Δ*=−0.46 (95% CI −0.90 to −0.03)	.03	Yes
AI vs fellows		*Δ*=−0.07 (95% CI −0.37 to 0.24)	.86	No
Residents vs fellows		*Δ*=0.40 (95% CI −0.05 to 0.85)	.09	No

#### Impression With Negative Impact

At last, 7 of 200 (3.5%) impressions were identified as potentially having a negative medicolegal impact on patient outcomes: 3 of 100 (3%) from AI, 1 of 26 (3.8%) from residents, and 3 of 74 (4.1%) from fellows. This low incidence finding was observed across all groups, with no statistically significant difference (*P*=.93). Figure 1A-E shows 100% stacked bar charts comparing the distribution of responses to Likert-scale questions.

### Thematic Analysis

#### AI vs Trainees Analysis

**Figure 2.**
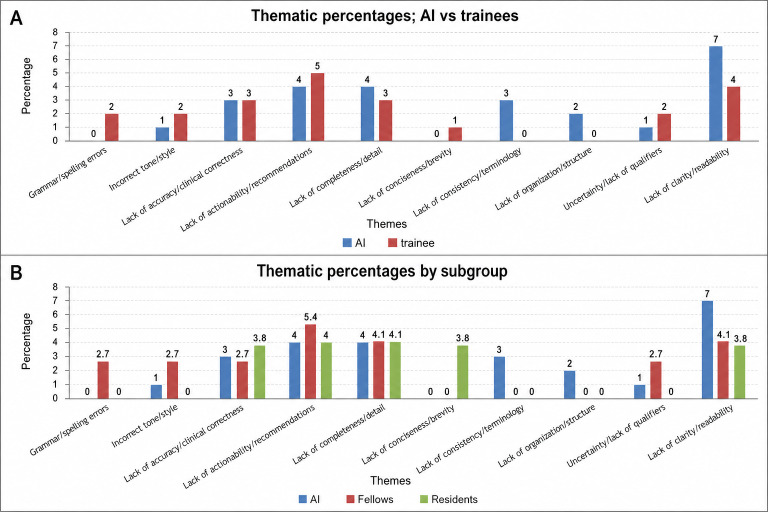
Thematic analysis summary figure of criticisms received. (A) AI vs trainees (top) and (B) subgroup of AI vs residents vs fellows (bottom). AI: artificial intelligence.

Thematic analysis allowed free-text comments of reviewers to be categorized and used to clarify aspects of performance not fully captured by the questionnaire. These results also help to better evaluate the performance of each group. The thematic scoring is detailed in Table 4 and is graphically summarized in Figure 2A.

**Table 4.** Thematic analysis summary of criticisms received by artificial intelligence (AI) vs trainees.

Theme	AI, n (%)	Trainee, n (%)	Fisher *P* value	Significance
Lack of accuracy/clinical correctness	3 (3)	3 (3)	>.99	Nonsignificant
Lack of actionability/recommendations	4 (4)	5 (5)	>.99	Nonsignificant
Lack of clarity/readability	4 (4)	3 (3)	>.99	Nonsignificant
Lack of conciseness/brevity	3 (3)	0 (0)	.04	Significant
Lack of consistency/terminology	2 (2)	0 (0)	.32	Nonsignificant
Lack of organization/structure	1 (1)	2 (2)	>.99	Nonsignificant
Incorrect tone/style	1 (1)	2 (2)	>.99	Nonsignificant
Uncertainty/lack of qualifiers	7 (7)	4 (4)	.046	Significant
Lack of completeness/detail	0 (0)	1 (1)	>.99	Nonsignificant
Grammar/spelling errors	0 (0)	2 (2)	.50	Nonsignificant

The comments were categorized into 10 themes: Lack of accuracy/clinical correctness; Lack of actionability/recommendations; Lack of conciseness/brevity; Lack of consistency/terminology; Lack of organization/structure; Incorrect tone/style; Uncertainty/lack of qualifiers; Lack of completeness/detail; Lack of clarity/readability; Grammar/spelling errors.

Using Fisher exact test across all themes, a total of 56 comments were received for 200 report samples. A qualitative review of open-ended evaluator comments showed that most categories of critique occurred infrequently and did not differ significantly between AI and trainees. Observations related to accuracy or clinical correctness (AI 3%, trainee 3%; *P*>.99), actionability or recommendations (AI 4%, trainee 5%; *P*>.99), and clarity or readability (AI 4%, trainee 3%; *P*>.99) were evenly distributed. Likewise, themes addressing impression organization, tone, terminology, consistency, completeness, or detail showed no meaningful differences. These findings suggest that, in most qualitative aspects, reviewer perceptions of AI-generated and trainee-generated impressions were broadly comparable.

Two themes demonstrated statistically significant group differences. AI-generated impressions were more frequently criticized for a lack of conciseness or brevity (AI 3% vs 0%; *P*=.04), indicating a tendency for longer and more detailed narrative structures. Additionally, AI reports were more often flagged for uncertainty or a lack of qualifying language (AI 7% vs 4%; *P*=.046), reflecting greater cautious phrasing in diagnostic statements. These observations suggest that while the overall quality of AI impressions was similar to trainee reports, reviewers perceived AI outputs as wordier and slightly more tentative in tone.

#### Subgroup Analysis (AI vs Residents vs Fellows)

When the thematic analysis was expanded to examine AI-generated, resident-generated, and fellow-generated impressions separately, the overall pattern of findings remained consistent with the 2-group comparison (Table 5).

Most critique categories, including accuracy, actionability, clarity, organization, tone, terminology, and completeness, occurred infrequently and did not differ significantly among the 3 subgroups. Minor clarity concerns appeared slightly more often in AI and fellow reports but were not statistically significant (*P*=.58). Grammar and spelling remarks were rare and confined to fellow impressions (*P*=.16).

**Table 5.** Thematic analysis summary of criticisms received by subgroup (N=200).

Theme	AI^a^^,^^b^, n (%)	Resident^c^, n (%)	Fellow^d^, n (%)	Approximate Fisher *P* value	Significance
Lack of accuracy/clinical correctness	3 (3)	1 (3.8)	2 (2.7)	.99	Nonsignificant
Lack of actionability/recommendations	4 (4)	1 (3.8)	4 (5.4)	.96	Nonsignificant
Lack of clarity/readability	4 (4)	0 (0)	3 (4.1)	.58	Nonsignificant/trend
Lack of conciseness/brevity	3 (3)	0 (0)	0 (0)	.047	Significant
Lack of consistency/terminology	2 (2)	0 (0)	0 (0)	.31	Nonsignificant
Lack of organization/structure	1 (1)	0 (0)	2 (2.7)	.60	Nonsignificant
Incorrect tone/style	1 (1)	0 (0)	2 (2.7)	.60	Nonsignificant
Uncertainty/lack of qualifiers	7 (7)	1 (3.8)	3 (4.1)	<.001	Significant
Lack of completeness/detail	0 (0)	1 (3.8)	0 (0)	>.99	Nonsignificant
Grammar/spelling errors	0 (0)	0 (0)	2 (2.7)	.16	Nonsignificant

a AI: artificial intelligence.

b n=100.

c n=26.

d n=74.

Two themes continued to demonstrate statistically significant variation across groups. AI reports were more frequently noted for a lack of conciseness or brevity (3.0% vs 0% for both residents and fellows, *P*=.047), reinforcing the perception that AI outputs tended to be more wordy. Uncertainty or lack of qualifiers was also more common in AI impressions (7.0% vs 3.8% for residents and 4.1% for fellows, *P*<.001), suggesting greater use of cautious phrasing. These findings indicate that, apart from being somewhat longer and more tentative in tone, AI-generated impressions were qualitatively similar to those produced by residents and fellows. Figure 2B is also a visual demonstration of the thematic analysis by subgroup.

## Discussion

### Clinical Implications of AI-Assisted Reporting

The results of this study highlight the advantages of AI-generated impressions in nuclear medicine compared to trainee-generated impressions, aligning with contemporary literature on AI-assisted diagnostic imaging [9-13]. Importantly, the observed improvements in completeness and clarity reflect enhanced synthesis of provided information rather than independent image-based diagnostic reasoning. AI-generated impressions in this cohort demonstrated superior completeness and a more consistent use of diagnostic certainty terminology—the 2 domains previously identified as sources of variability and potential clinical error in nuclear medicine reporting. Although statistically significant, these differences were associated with small effect sizes, suggesting modest clinical impact that should be interpreted with caution. AI-assisted reporting depends on the quality and completeness of the input data, and omissions in the report stem may be propagated rather than corrected. The AI model was provided with structured report inputs, which differ from real-world trainee workflows and may have contributed to improved completeness and standardized language. Standardized diagnostic language is instrumental in communicating risk, guiding downstream decisions, and reducing the likelihood of misinterpretation by referring physicians [14-17].

Clarity and correctness were found to be comparable between AI-generated and trainee-generated impressions, a finding echoed in studies evaluating the quality of AI-generated reports against human experts in radiology and nuclear medicine [18]. While AI systems deliver structured and highly reproducible outputs, human-generated reports, especially those authored by more experienced trainees, still provide nuanced clinical context—an aspect where AI sometimes underperforms due to its reliance on template-driven logic and a lack of experience-driven intuition [19,20]. The lack of brevity in this study may be mitigated through prompt optimization, including explicit length constraints and structured templates to balance completeness with conciseness.

Notably, a subgroup analysis in this study indicated that international trainees with previous nuclear medicine training outperformed radiology residents who were native English speakers but had less experience in nuclear medicine PET/CT imaging in terms of report completeness and clarity, suggesting that exposure remains a critical lever for report quality. AI may be beneficial for less experienced trainees, helping them improve quickly and reducing mistakes or missing information by supporting their learning.

Other studies also support this, with AI integration shown to boost efficiency, reduce addendum rates, and streamline patient triage [21,22]. However, there is a need for continued oversight and physician presence in AI-generated reports to mitigate potentially negative management impacts. Future implementations should ensure that AI report provenance is clear, outputs are subject to faculty sign-off, and safeguards are in place for rare or complex cases. Prospective, multicenter studies should also evaluate clinical outcomes related to AI-assisted reporting, including diagnostic yield, time to diagnosis, and medicolegal parameters [11,13,23].

In this blinded comparison of AI-generated and trainee-generated PET/CT impressions, AI achieved noninferior correctness and clarity while demonstrating advantages in completeness and consistent use of certainty terminology. Several evaluated outcomes (eg, clarity, completeness, and satisfaction) are inherently subjective, and although interrater agreement was substantial, this subjectivity may influence the interpretation of performance differences. These findings suggest that structured AI outputs can address 2 common challenges in trainee reporting, including omissions and inconsistent phrasing without sacrificing accuracy. The higher frequency and level of certainty terms in AI impressions are notable. The use of certainty modifiers carries meaningful clinical implications in diagnostic communication.

In this study, AI-generated impressions contained certainty words more frequently and tended to express higher degrees of diagnostic confidence compared with trainee reports (Table 1; *P*=.02). Excessive omission of such terms in trainee reports can lead to ambiguous or overly cautious interpretations, potentially limiting clinical decisiveness or delaying management decisions. Conversely, overly assertive wording without appropriate qualification may create false diagnostic confidence and potentially contribute to inappropriate clinical decision-making. Maintaining calibrated, probability-based terminology (eg, “likely ~75%” and “consistent with >90%”) helps align radiologic communication with clinical reasoning and supports reproducible decision-making.

Our findings suggest that structured AI phrasing may enhance clarity and interpretive confidence, but appropriate contextual use of certainty words remains essential to balance precision with caution in diagnostic reporting. Standardized diagnostic language helps communicate probability and clinical confidence, which is central to downstream decision-making [24]. Our results align with literature describing AI’s capacity to reduce variability and improve report standardization in radiology [24]. Recent work has also emphasized the importance of robust evaluation frameworks for LLM outputs in clinical contexts [25]. Emerging evidence also supports the role of LLMs in imaging-related clinical interpretation workflows [26]. By contrast, trainees’ strengths lie in case-specific details and adaptive reasoning, which are qualities that remain essential for complex or atypical presentations. Subgroup results provide important context that international trainees (with ≥1 y of nuclear medicine training) outperformed Canadian radiology residents (with limited nuclear medicine exposure) in completeness and, numerically, clarity. This supports the interpretation that training duration and exposure are key determinants of report quality. It also underscores how AI might be most beneficial for earlier-stage trainees or in rotations where exposure is limited, by providing consistent scaffolding and a didactic template. Clinically, more correctness (92%) and complete impressions (97%) in AI-generated impressions, along with explicit action plans (67/81, 82.7%) compared to trainee-generated reports (91%, 92%, and 59%, respectively), would reduce ambiguity and help standardize care pathways by prompting appropriate next steps (eg, histopathology or additional imaging). Nevertheless, 3% of reviews signaled potential negative impacts in AI-generated impressions though still below the negative impacts of trainee-generated impressions, highlighting the need for human oversight. AI-generated impressions may use generic lexicon or be too wordy, but giving the AI more specific instructions or using locally designed templates can help make AI outputs more precise and relevant.

In conclusion, the findings of this study support a model in which AI further improves the quality of the impression section of the PET/CT report, particularly among less experienced trainees with limited expertise in nuclear medicine reporting. Carefully designed AI templates and locally tailored instructions may provide a practical pathway to improving impression quality, supporting trainee education, and ultimately enhancing patient care. The use of AI in diagnostic reporting also raises ethical considerations regarding accountability, as the ultimate responsibility for interpretation and clinical decisions remains with the reporting physician.

### Limitations

This single-center study used a moderate sample size (200 impressions) and a single AI model (ChatGPT-4o) with a fixed prompt and lexicon. Results may differ with other models, other sorts of prompt designs, templates, or clinical settings. Although raters were blinded, stylistic cues might have partially unblinded the source. The case mix was limited to PET/CT studies in one institution and did not include outcome-linked end points; thus, clinical impact is inferred rather than demonstrated. In this study, the trainees had access to full information (pathology, prior images, treatments, clinical information, etc), while AI and raters assessed impressions only from report stems. This constitutes a limitation and should be acknowledged. The difference in information access may have influenced comparative performance and should be considered when interpreting the results. A key limitation of this study is that AI-generated impressions were based solely on the written report components (clinical history, technical details, and findings/stem) and did not incorporate direct image interpretation. Therefore, this study evaluates the linguistic quality, structure, and consistency of report impressions rather than true diagnostic accuracy relative to imaging data. As such, the findings should be interpreted as reflecting improvements in report formulation and usability rather than the independent diagnostic capability of AI systems.

Interrater reliability and the possibility of improving agreement could be further explored through studies investigating sources of interrater disagreement, including whether specific lesion types or clinical scenarios contribute to variability in scoring. Finally, subgroup analyses (residents vs fellows trainees) may be influenced by unmeasured confounders (eg, prior report volume, language proficiency, or subspecialty focus).

### Future Directions

Prospective, multicenter trials should evaluate workflow integration (time-to-report, addendum rates), downstream clinical outcomes (diagnostic yield of recommended actions, time-to-treatment), and medicolegal dimensions (consistency, error rates, and escalation triggers). Research should test adaptive AI systems that learn local style guides and incorporate structured reporting templates and certainty lexicons. Educational studies should determine how AI assistance influences trainee learning curves, autonomy, and feedback quality. Transparent frameworks are needed to define responsibilities, auditing, and disclosure of AI assistance in clinical documentation. Finally, emerging approaches, such as physics-informed AI that integrates quantitative imaging biomarkers (eg, standardized uptake values) with language models, may further enhance the clinical utility of AI-assisted reporting. Given the similar reporting structure across nuclear medicine studies, this approach may extend to other areas; however, dedicated validation in different modalities and clinical settings is warranted.

### Conclusions

AI-generated PET/CT impressions, based on the provided report text (without direct image input), were comparable to trainee impressions in correctness and clarity and were superior in completeness, consistent use of certainty terminology, and in providing action plans when appropriate. International fellow trainees outperformed Canadian residents, supporting the role of prior nuclear medicine experience in report quality. Together, these results indicate that AI can function as a complementary tool for standardizing language, reinforcing completeness, and prompting explicit next steps in the impression of the report, while faculty oversight preserves clinical correctness and safeguards against generic or misleading statements. Despite improvements in completeness and structure, the similar rate of potential negative medicolegal impact suggests no clear advantage in core diagnostic safety in this academic setting; however, variability across practice environments means even small reductions may be meaningful, reinforcing AI as an assistive rather than replacement tool. Adoption pathways should prioritize education, local customization of prompts and lexicons, and thorough evaluation of clinical and operational outcomes.

## Supplementary material

10.2196/94833 Multimedia Appendix 1 Questionnaire and a list of certainty words.

## Abbreviations

AI: artificial intelligence
CT: computed tomography
LLM: large language model
PET: positron emission tomography
