# Supplementary material for: Exploring the Role of AI in Enhancing Nuclear Medicine Report Impressions Generated by Trainees and ChatGPT-4o: Comparative Evaluation Study
Source: JMIR AI. 2026 Jul 8;5:e94833. doi: 10.2196/94833 (PMC13344531; doi:10.2196/94833)
Supplement: Multimedia Appendix 1 [file ai-v5-e94833-s001.docx]

**Questionnaire Case No.**

| The information is  relevant score=1 | Partially covered all findings score= 0 | Inaccurate impression score= -1 |
| --- | --- | --- |

Impression can have a negative impact on patient management. Yes No

(not providing relevant information, follow-up plan, or further imaging)

An action plan is provided. Yes No NA
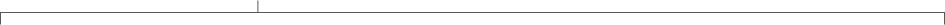
 Further imaging Histopathology correlation Close follow-up Clinical Consult

At least one certainty word used in the impression? Yes No

What kind of certainty word is used in the impression? (Mark as many as applicable, use Table 2 as a reference)

5=Highest 4=High 3=Intermediate 2=Low 1=Very low 0=None

**Note: In biopsy-proven cases, the impression should reflect high/est certainty word. If it does not, mark it as 0=none)**

| **Completely agree=5** | **Agree=4** | **Average=3** | **Partially agree=2** | **Completely disagree=1** |
| --- | --- | --- | --- | --- |

The report impression was clear.

The report impression was complete.

The impression was well-organized.

| **5=Very**  **satisfied** | **4=Satisfied** | **3=Average** | **2=Dissatisfied** | **1=Very**  **dissatisfied** |
| --- | --- | --- | --- | --- |

Overall satisfaction with the impression

Comments: Please provide any additional comments regarding this impression beyond the points mentioned above.
